# Redefine the Role of Spot-Scanning Proton Beam Therapy for the Single Brain Metastasis Stereotactic Radiosurgery

**DOI:** 10.3389/fonc.2022.804036

**Published:** 2022-05-19

**Authors:** Sheng Chang, Gang Liu, Lewei Zhao, Weili Zheng, Di Yan, Peter Chen, Xiangpan Li, Kunyu Yang, Rohan Deraniyagala, Craig Stevens, Inga Grills, Prakash Chinnaiyan, Xiaoqiang Li, Xuanfeng Ding

**Affiliations:** ^1^ Department of Radiation Oncology, Renmin Hospital, Wuhan University, Wuhan, China; ^2^ Department of Radiation Oncology, Beaumont Health System, Royal Oak, MI, United States; ^3^ Cancer Center, Union Hospital, Tongji Medical College, Huazhong University of Science and Technology, Wuhan, China

**Keywords:** single brain metastasis, stereotactic radiosurgery, spot-scanning, proton arc therapy, intensity modulated proton therapy, volumetric modulated arc therapy, brain radionecrosis

## Abstract

**Purpose:**

To explore the role of using Pencil Beam Scanning (PBS) proton beam therapy in single lesion brain stereotactic radiosurgery (SRS), we developed and validated a dosimetric in silico model to assist in the selection of an optimal treatment approach among the conventional Volumetric Modulated Arc Therapy (VMAT), Intensity Modulated Proton Therapy (IMPT) and Spot-scanning Proton Arc (SPArc).

**Material and Methods:**

A patient’s head CT data set was used as an in *silico* model. A series of targets (volume range from 0.3 cc to 33.03 cc) were inserted in the deep central and peripheral region, simulating targets with different sizes and locations. Three planning groups: IMPT, VMAT, and SPArc were created for dosimetric comparison purposes and a decision tree was built based on this in *silico* model. Nine patients with single brain metastases were retrospectively selected for validation. Multiple dosimetric metrics were analyzed to assess the plan quality, such as dose Conformity Index (CI) (ratio of the target volume to 100% prescription isodose volume); R50 (ratio of 50% prescription isodose volume to the target volume); V_12Gy_ (volume of brain tissue minus GTV receiving 12 Gy), and mean dose of the normal brain. Normal tissue complication probability (NTCP) of brain radionecrosis (RN) was calculated using the Lyman-Kutcher-Burman (LKB) model and total treatment delivery time was calculated. Six physicians from different institutions participated in the blind survey to evaluate the plan quality and rank their choices.

**Results:**

The study showed that SPArc has a dosimetric advantage in the V_12Gy_ and R50 with target volumes > 9.00 cc compared to VMAT and IMPT. A significant clinical benefit can be found in deep centrally located lesions larger than 20.00 cc using SPArc because of the superior dose conformity and mean dose reduction in healthy brain tissue. Nine retrospective clinical cases and the blind survey showed good agreement with the in *silico* dosimetric model and decision tree. Additionally, SPArc significantly reduced the treatment delivery time compared to VMAT (SPArc 184.46 ± 59.51s vs. VMAT: 1574.78 ± 213.65s).

**Conclusion:**

The study demonstrated the feasibility of using Proton beam therapy for single brain metastasis patients utilizing the SPArc technique. At the current stage of technological development, VMAT remains the current standard modality of choice for single lesion brain SRS. The *in silico* dosimetric model and decision tree presented here could be used as a practical clinical decision tool to assist the selection of the optimal treatment modality among VMAT, IMPT, and SPArc in centers that have both photon and proton capabilities.

## Introduction

Stereotactic Radiosurgery (SRS) is a non-invasive alternative treatment method for brain metastases (BM) (1, 2). It can be delivered through several modalities, such as Gamma Knife, Cyberknife, conventional radiotherapy linear accelerators (linac-based SRS), or passive-scattering proton beam therapy (3–5). Brain radionecrosis (RN) is one of the major side effects. It was reported that this long-term complication had been linked to V_12Gy_ (the volume of healthy brain tissue irradiated with 12 Gy) of the brain tissue (6–8). Some studies suggested that keeping V_12Gy_ below 8.5 cc could reduce the risk of brain RN (5, 7, 9). Current practice in many centers is to follow stepwise prescription schemes according to the size of the lesion, with generally lower doses for larger lesions (10–14). Importantly, local control is highly dependent on the prescribed dose and negatively associated with target volume in photon therapy due to the significant exit dose (5, 11–13). The ability to deliver ablative doses of radiation, particularly to patients with large brain tumors, is often limited by this constraint to spare an adequate volume of normal brain. For a target diameter of more than 3 cm, it has been recommended to use fractionated treatment other than single-fraction SRS to mitigate the radiation-induced toxicity (15, 16).

Proton beam therapy offers the potential clinical benefit to further spare healthy brain tissue by taking advantage of its unique physical characteristic, “Bragg Peak,” in which the rapid dose fall-off offers zero dose beyond the target’s distal edge. The pencil beam scanning (PBS) technique, which delivers the proton treatment *via* spot by spot and energy layer by layer, significantly improves the dose conformity at the proximal region compared to the passive-scattering technique (17, 18). Recently, such treatment methodology has been adopted by most of the new proton therapy centers (19). However, due to the large in-air spot size, the PBS technique has a much larger lateral penumbra than the passive-scattering technique or photon radiotherapy techniques such as IMRT or VMAT (20, 21). This critical physics parameter limits its clinical implementation in the single fractionation brain SRS where a sharp gradient dose fall-off is desired to protect adjacent healthy tissue or organs (22–24). To our best of knowledge, there is no report of using PBS for brain SRS to date. Thus, there is an immediate need to address this challenge and continue to develop the PBS technique to meet such clinical needs.

In 2016, Spot-scanning proton arc (SPArc) therapy was proposed by Ding et al. to improve the dosimetric plan quality of PBS while making the arc therapy efficient, robust, and compatible with the current PBS technique without major hardware modifications (25). This new concept has recently been integrated into an existing clinical system as the prototype proton arc machine (26). Previous studies have demonstrated its potential clinical benefits in the conventional treatment fraction for head and neck, brain, prostate, lung, spine, and breast cancer patients, compared to the IMPT (27–33). However, no studies have been conducted to exploit the potential dosimetric benefits and feasibility in brain SRS. We hypothesized that by taking advantage of the degree of freedom through arc(s) trajectory, SPArc might have the flexibility and the optimization freedom to balance the sharp distal fall-off and larger lateral penumbra to provide an optimal dosimetric plan quality and treatment solution for a single target brain SRS compared to the conventional VMAT and IMPT. We aimed to build a dosimetric prediction model through an *in silico* planning study with a variety of tumor sizes and locations compared among the SPArc, IMPT, and VMAT plans. The validation tests were then performed through clinical patient datasets previously treated by single-fraction (SSRT) and fractionated stereotactic radiotherapy (FSRT) and then followed by a blind survey of clinicians worldwide. To our best knowledge, this is the first comprehensive study to build an *in silico* dosimetric model to assist the clinical decision-making among three treatment modalities, including IMPT, VMAT, and SPArc for single BM SRS.

## Methods And Materials

### *In Silico* Brain SRS Dosimetric Model

**Figure 1:**
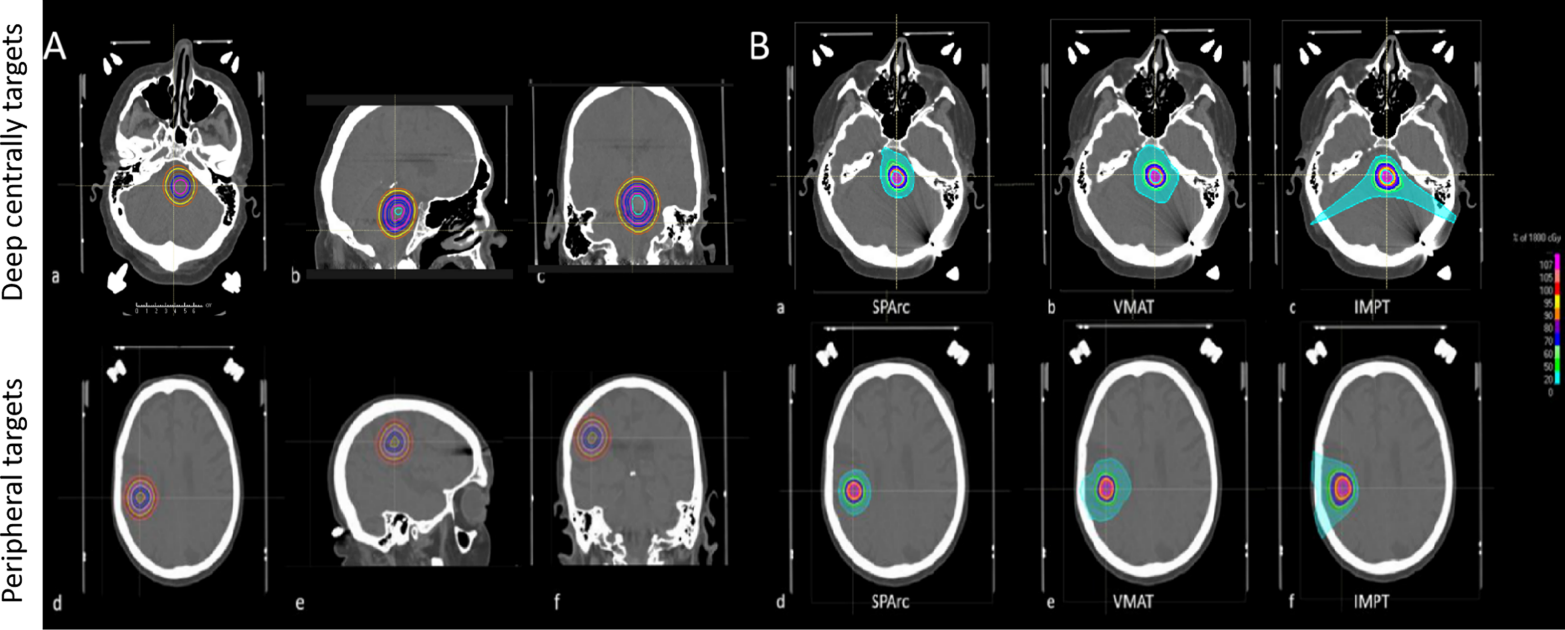
**(A)** Target’s locations and sizes (from 0.3cc to 33.03cc) in the brain SRS dosimetric model. Deep centrally located targets (a, b, c); Peripheral targets (d, e, f). **(B)** A representative transverse view of dose distributions among SPArc, IMPT, and VMAT on the 0.3cc target. Deep centrally located 0.3cc target (a) SPArc, (b) VMAT, (c) IMPT; Peripheral 0.3cc target (d) SPArc, (e) VMAT, (f) IMPT.

A patient’s CT image and structure set were used as an *in silico* head phantom to develop a brain SRS dosimetric model to assist the optimal treatment modality selection. A spherical-shaped target, Gross Tumor Volume (GTV) (0.3 cc), was inserted in the deep central and peripheral region of the CT. The deep central targets are located at a depth of 5.65 cm from the brain surface and the peripheral region targets are located at a depth of 1.06 cm from the brain surface. The GTV was then expanded with a uniform margin every 2mm increments, corresponding to a different target volume (from 0.3 cc to 33.03 cc) (**Figure 1**). The target volume extending outside of the brain structure was excluded. Three treatment modalities IMPT, VMAT, and SPArc were generated in Raystation ver. 9A using the same planning robust optimization parameters (2mm setup and 3.5% range uncertainty for proton planning and 2mm setup uncertainty for VMAT planning). Each VMAT plan consisted of two coplanar and two non-coplanar arcs with 6MV photon. The coplanar arcs were rotated clockwise from 181°–179° and rotated counterclockwise from 179°–181°, two non-coplanar arcs were placed at couch angles of 45° and 315°. The SPArc plan consisted of one coplanar and two non-coplanar arcs. The couch positions and arc rotations of the two non-coplanar arcs were the same as those of VMAT, while the 3-field IMPT was delivered with two posterior oblique fields along with a vertex field (Gantry angle of 90 and couch angle of 270) (**Table S1**). Range shifter was used in the IMPT plan but not in the SPArc plan due to the sufficient degree of freedom. For SPArc, three arcs were used with a sampling frequency of 2.5° per control point. In other words, the SPArc plan consists of a total of 288 beam angles. Both SPArc and IMPT used the same physics beam model based on IBA ProteusONE^®^ with an energy range from 70MeV to 227.7MeV. For more beam specific parameters, the study used RayStation’s default setting such as the automatic energy layer spacing and spot spacing for optimization in both the IMPT and SPArc planning groups (**Figure S1**). The prescription was 18 Gy (RBE) in 1 fx, with at least 96% of GTV receiving a full prescription dose in the worst-case scenario robustness evaluation. Multiple dosimetric metrics were analyzed to assess the plan quality, such as dose Conformity Index (CI) (ratio of the target volume to 100% prescription isodose volume); R50 (atio of 50% prescription isodose volume to the target volume); V_12Gy_ (Volume of normal brain tissue irradiated with at least 12 Gy); and mean dose of the normal brain. The normal brain tissue was defined as brain tissue minus GTV.

### Clinical Validation Tests

To validate the *in silico* brain SRS model, nine patients with single brain metastasis previously treated by single or multi-fraction SRS at our institution were retrospectively included in the study. The target volumes and previous clinical prescribed doses (Gy) and fraction are detailed in **Table 1** for each of the nine patients, in which two patients received fractionated brain SRS (FSRT) due to the size and location of the lesion. All CT-data sets with corresponding structure contours were transferred from gamma plan™ to Raystation 9A and replanned with IMPT, VMAT, and SPArc with prescription 18 Gy in one fraction. These nine cases were separated into two groups based on the location: four deep central and five peripheral located targets. The GTVs volume ranges from 1.66 cc–28.65 cc. The target CI, R50, mean dose of the brain, and V_12Gy_ of the cases were analyzed and compared to the brain SRS dosimetric model (**Table 2**). We generated a brain SRS decision tree based on tumor size and location using the *in silico* model.

**Table 1:** Target size and previous clinical prescription for the 9 patients included in this SRS study comparison.

Patient	Tumor location	Tumor volume (cc)	Previous Clinical prescription dose and fraction
#1	deep central region	1.66	21Gy/1f
#2	peripheral region	3.53	21Gy/1f
#3	peripheral region	14.65	15Gy/1f
#4	peripheral region	4.13	18Gy/1f
#5	peripheral region	8.34	18Gy/1f
#6	deep central region	11.04	15Gy/1f
#7	deep central region	20.76	24Gy/3f
#8	peripheral region	24.70	15Gy/1f
#9	deep central	28.65	21Gy/3f

### Normal Tissue Complication Probability of Brain Necrosis

In this study, the normal brain was defined as the whole brain minus the GTV, and we evaluated the normal tissue complication probability (NTCP) of brain necrosis by using the Lyman-Kutcher-Burman (LKB) model (34) The single-fraction doses were converted to equivalent 2 Gy per fraction total doses using the linear-quadratic model assuming an α/β ratio of 2 Gy for normal tissue (35).


(1) $$\text{NTCP}=\frac{1}{\sqrt{2\pi }}\int_{-\infty }^{\text{t}}e^{-\frac{x^{2}}{2}}dx$$

(2) $$\text{t}=\frac{D-TD_{50}}{m\times TD_{50}}$$

where TD_50_ is the tolerance dose for a 50% complication probability for uniform doses to the organ and m is a dimensionless parameter for determining the slope of the complication probability according to the dose curve. For the uniform dose D, we used the generalized equivalent uniform dose (gEUD), as shown in:


(3) $$\text{gEUD}={\left(\sum_{i=1}^{N}V_{i}D_{i}^{a}\right)}^{\frac{1}{a}}$$

where Di is the dose for each bin in a differential dose–volume histogram (DVH), vi is the volume in a specific dose bin i, and N is the unequal fractional sub-volume. The ‘a’ value is a parameter equal to 1/n, in which n represents the volume dependence of the complication probability. We adopted the following parameters to evaluate the radiation-induced brain necrosis as an endpoint: TD50 = 60, m = 0.15, a = 4. In radiosurgery, the dose is delivered in a single fraction. The single-fraction doses were converted to equivalent 2 Gy per fraction total doses using the biologically effective dose (BED) formalism of the linear-quadratic model assuming an α/β ratio of 2 Gy for normal tissue and 10 Gy for tumor tissue [36]. The formula used to calculate EQD2 was:


(4) $$\text{EQD}2=N\times d\times \frac{\text{d}+\text{α}/\text{β}}{2+\text{α}/\text{β}}$$

with N = number of fractions, d = dose, α = linear coefficient reflecting cellular radiosensitivity, and β = quadratic coefficient reflecting cell repair mechanisms.

### Tumor Control Probability and Dose De-Escalation Study

To explore the TCP and NTCP relationship in the challenge case (patient #7 and #9) in which single fraction might not be safe due to the risk of RN, a series of dose de-escalation plans were performed using VMAT and IMPT. The corresponding TCP was accessed based on the logistic model as following:


(5) $$TCP\left(\left\{D_{i}\right\}\right)={\prod_{i=1}^{N}\left[\frac{1}{1+{\left(\frac{D50}{D_{i}}\right)}^{4*\gamma 50}}\right]}^{\frac{1}{N}}$$

N is the total number of voxel in tumor, each voxel receiving a uniform dose. D50 denotes the dose to control 50% of tumors, and ɤ_50_ is the relative slope of the TCP curve at D50, which are 27.04 Gy and 0.75, respectively (36, 37).

### Treatment Beam Delivery Time Calculation

The treatment delivery efficiency of the IMPT and SPArc plans was evaluated based on a proton system with gantry rotation max speed 6 deg/s, 2 ms spot switching time and ELST 0.7s (25). The VMAT plans times were simulated based on the Elekta Versa HD with a dose rate of 600 MU/min and 1,400 MU/min with flattening-filter-free (FFF) beams with 6X photon beams energy and gantry rotation max speed 6 deg/s.

### Data and Statistical Analysis

Treatment plan metrics, NTCP, and treatment delivery time among IMPT, VMAT, and SPArc were compared with a paired, two-tailed nonparametric Wilcoxon signed-rank test using SPSS 21.0 software (International Business Machines, Armonk, New York). P values of less than 0.05 were considered statistically significant. All the tests were performed with VMAT as a reference.

### Plan Evaluation and Survey Among the Physician Group

To test if the *in silico* model is able to provide useful clinical guidance in the selection of optimal treatment modality, the nine clinical cases from the retrospective study (described in the previous section) were sent to six physicians from different institutions worldwide. To avoid any preference or bias during the plan dosimetric evaluation, the name of each plan was masked as #a, #b, and #c as a blind survey. Only target coverage, CI & R50, mean dose of the brain, and V_12Gy_ along with the 3D dose distribution were presented. Each physician ranked each plan from 1–3 (1: the most preferred choice; 2: the intermediate choice; 3: the least preferred choice) based on their clinical experience. To mitigate other factors that may impact the clinical decision other than dosimetric plan quality, all the physicians were informed that the patients’ diagnosis is single brain metastasis from an unknown primary tumor. The patients were in the mid 50s age range and expected to live five years after the treatment. A sample of the survey was included in the supplemental document. Then, the result of the blind plan evaluation survey was unmasked and analyzed.

## Results

### Brain SRS Dosimetric Comparison and Decision Tree

**Figure 2:**
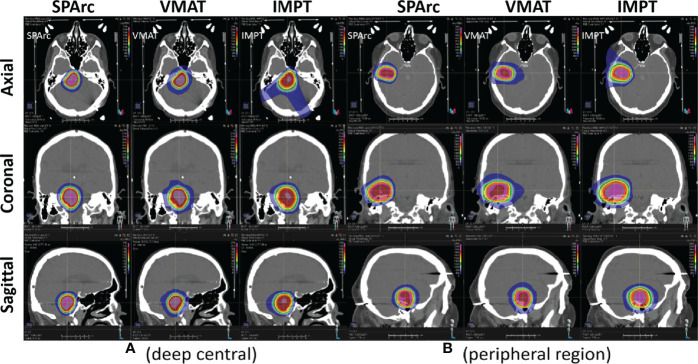
Representative SPArc, VMAT, and IMPT dose distributions for patients with deep central **(A)** and peripheral region **(B)** tumors in transversal, coronal, and sagittal planes. The threshold of 18 Gy was used to display dose distributions (color wash overlay).

**Figure 3:**
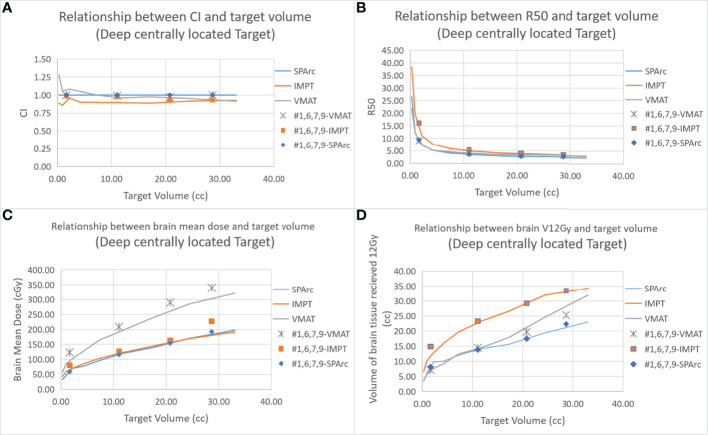
Dosimetric metrics among three planning groups: SPArc, VMAT, and IMPT at deep central **(A–D)** of different target sizes. Dots, squares, and stars are the dosimetric metrics extracted from four clinical validation cases normalized to SPArc plan (**Table 2**).

**Figure 4:**
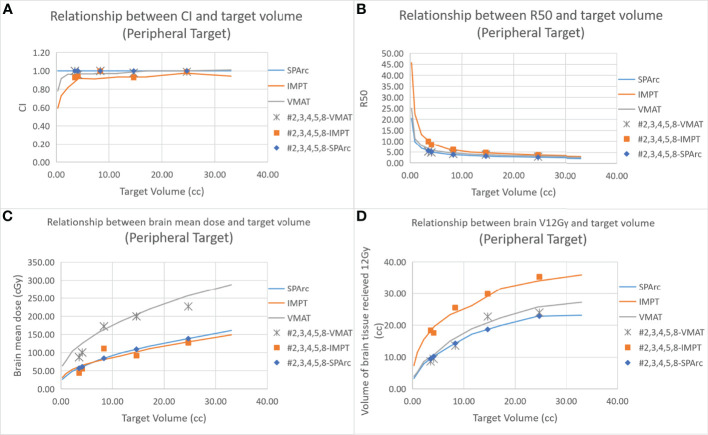
Dosimetric metrics among three planning groups: SPArc, VMAT, and IMPT at peripheral region **(A–D)** of different target sizes. Dots, squares, and stars are the dosimetric metrics extracted from five clinical validation cases normalized to SPArc plan (**Table 2**).

**Figure 5:**
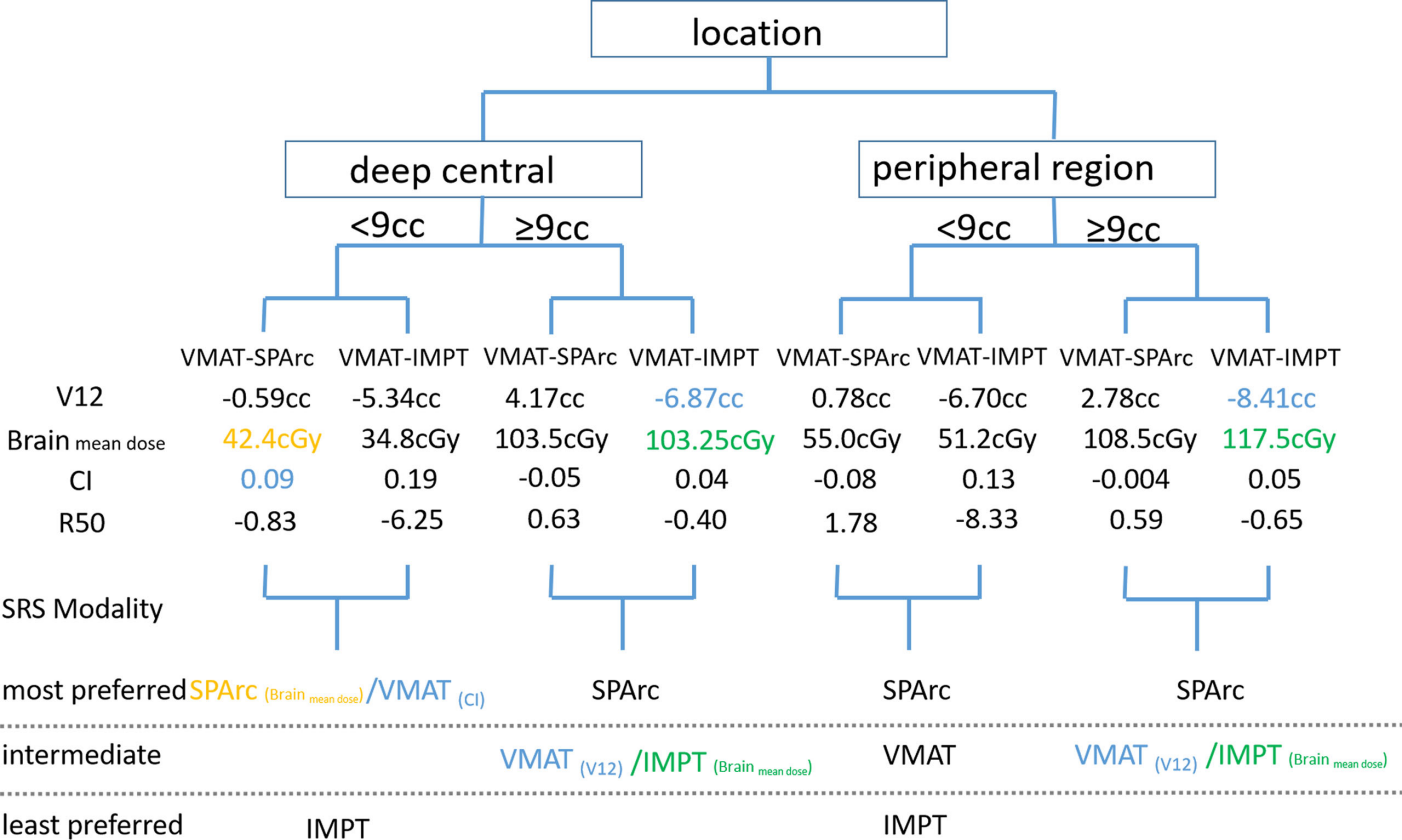
Treatment decision tree and model to predict the optimal treatment modality based on tumor size and location. SPArc would be the optimal choice in most of the target size or location except deep centrally located target. This would be the physician’s clinical decision to favor the CI or brain mean dose sparing.

**Figure 2** shows an example of SPArc, VMAT, and IMPT dose distributions for patients with deep central (A) and peripheral region (B). Each dosimetric metric, such as CI, R50, mean dose of the brain, and V_12Gy_ was plotted as a function of the target size in the deep central (**Figure 3**) and peripheral regions (**Figure 4**). Compared to IMPT, VMAT showed its significant advantage in the CI and R50 in any target size less than 30cc in peripheral and deep central locations and V_12Gy_ to the brain. Conversely, SPArc has an equivalent or better CI in any size of peripheral targets and the deep centrally located targets bigger than 9cc compared to VMAT. For the deep centrally located tumor smaller than 9cc, the VMAT plan still offered better dose CI and V_12Gy_. With the advantage of proton beam characteristics, both SPArc and IMPT significantly reduced brain mean dose by nearly 2-fold compared with VMAT. The SPArc plan would be favored in most cases except deep central located target (< 9 cc) where VMAT shows a slight improvement over SPArc in CI, R50, and V_12Gy_ of the brain (**Figure 5**).

### Clinical Validation Tests

The results showed good agreement between the *in silico* model and the clinical cases (GTV range: 1.66 - 28.65 cc). The SPArc plans spared a significant volume of the normal brain compared to VMAT plans in all five patients with tumors > 9cc at any location (1.83 ± 1.15 cc) (**Figures 3**, **4**, and **Table 2**). Both the SPArc and IMPT planning groups reduced the mean brain dose delivered to all patients by 44.25% ± 5.70% (p < 0.01) and 38.44% ± 6.34% (p < 0.01) compared to the VMAT planning group. As predicted from the model, the VMAT plan offered slightly better V_12Gy_ (3.55 cc) compared to SPArc (4.06 cc) for patient # 1 with tumor < 9cc at the deep central location. For the three patients with tumor < 9 cc in the peripheral region, the VMAT plan provided a comparable V_12Gy_ compared to SPArc and IMPT. In terms of the delivery time (including irradiation time, gantry rotation, and couch rotation), the VMAT and VMAT (FFF) plans took much longer to deliver than any proton-based radiosurgery plan (**Table 3**). The mean treatment delivery time in the VMAT/VMAT(FFF) plans was 1574.78 ± 213.65 s and 940.89 ± 102.56 s compared with SPArc (260.29 ± 41.74 s) (p < 0.01) and IMPT (184.46 ± 59.51 s, p < 0.01).

**Table 2:** Validation of optimal brain sparing modality based on tumor size and location for 9 patients.

Patient	Tumor location	Tumor volume(cc)	CI			R50					V_12Gy_(brain) (cc)					Mean dose(brain) (cGy)				
			VMAT	IMPT	SPArc	VMAT	Relative to SPArc(model)	IMPT	Relative to SPArc(model)	SPArc	VMAT	Relative to SPArc(model)	IMPT	Relative to SPArc(model)	SPArc	VMAT	Relative to SPArc(model)	IMPT	Relative to SPArc(model)	SPArc
1	deep central	1.66	1.00	0.99	1.00	5.09	8.97	9.15	16.12	5.34	3.55	7.10	7.43	14.87	4.06	77.00	122.97	50.00	79.85	37.00
2	peripheral region	3.53	1.00	0.93	1.00	4.75	5.21	9.07	9.94	5.11	6.21	8.63	13.23	18.38	6.81	81.00	87.62	41.00	44.35	53.00
3	peripheral region	14.65	0.98	0.93	1.00	3.20	3.81	4.05	4.82	2.62	12.62	22.71	16.61	29.89	10.38	148.00	199.87	68.00	91.83	81.00
4	peripheral region	4.13	0.96	0.94	1.00	4.07	4.77	7.17	8.40	4.31	6.57	9.46	12.20	17.57	7.07	79.00	100.58	44.00	56.02	48.00
5	peripheral region	8.34	1.00	1.00	1.00	4.63	4.12	6.84	6.09	4.29	17.42	13.56	32.77	25.51	18.37	161.00	172.33	104.00	111.32	79.00
6	deep central	11.04	0.99	0.98	1.00	3.60	4.00	4.84	5.37	3.17	13.11	14.46	21.17	23.34	12.56	167.00	208.53	102.00	127.37	93.00
7	deep central	20.76	0.97	0.94	1.00	2.93	3.29	3.53	3.96	2.44	21.00	19.75	31.04	29.19	18.60	226.00	289.62	127.00	162.75	120.00
8	peripheral region	24.7	0.99	0.99	1.00	2.71	2.85	3.48	3.66	2.48	15.78	23.96	23.28	35.35	15.05	180.00	227.05	101.00	127.40	110.00
9	deep central	28.65	1.00	0.93	1.00	2.90	3.08	3.03	3.22	2.39	27.99	25.25	37.02	33.40	24.76	343.00	339.78	229.00	226.85	194.00

CI, Conformity Index; (Ratio of the target volume to 100% Prescription isodose volume).

R50, (Ratio of 50% Prescription isodose volume to the target volume).

V_12_Gy, (Volume of brain tissue minus GTV receiving12 Gy).

Relative to SPArc (model), (Ratio of SPArc plan between the model and case) × (absolute dosimetric metrics).

**Table 3:** Comparison of total delivery time per patient’s SRS plan.

Delivery time (Patient#)	VMAT (600 MU/min, s)	VMAT [1-5] (FFF, 1400 MU/min, s)	IMPT (s)	SPArc (s)
1	1635	808	85	207
2	1340	851	128	240
3	1516	972	206	288
4	1369	807	141	217
5	1946	1012	193	251
6	1809	1034	172	238
7	1631	1034	214	259
8	1605	1061	275	313
9	1322	889	244	328
Average	1574.78 ± 213.65	940.89 ± 102.56	184.46 ± 59.51	260.29 ± 41.74
P	–	<0.01*	<0.01*	<0.01*

*P < 0.05 while comparing the VMAT plan with other two plans.

NTCP of brain RN for each patient is listed in **Table 4**. All the NTCP of brain RN is clinically acceptable with 18Gy single fraction prescription doses (< 1%) except patients #7 and #9. More importantly, this study also found that the target location plays a critical part in the probability of brain RN as the peripheral target has much less EUD than the deep central located target in VMAT planning group. As a result, based on the VMAT technique, the NTCP of brain RN in patient #3 (peripheral target, volume 14.65 cc) and patient #8 (peripheral target, volume 24.70 cc) is 0.03% and 0.13%, respectively in comparison with patient #7 (deep central located target, volume 20.76 cc) 15.62% and patient #9 (deep central located target, volume 28.65 cc) 99.95%, respectively. In contrast, the probability of brain RN of patients #7 and #9 is 0.00% and 0.65% using the SPArc technique, indicating a superior dosimetric plan quality compared to the VMAT. Dose de-escalation for patients #7 and #9 was investigated from 1800cGy to 1000cGy (**Table 5**). The single fraction dose in VMAT had to be reduced to 1500cGy (#7) and 1000cGy (#9), respectively, in order to achieve an probability of RN (<1%).

**Table 4:** Normal tissue complication probabilities (%) calculated using a LKB model based on the Burman et al. Tolerance data.

Patient	Tumor location	Tumor volume (cc)	Normal tissue complication probabilities (%)		
			VMAT	IMPT	SPArc
1	deep central region	1.66	0.00	0.00	0.00
2	peripheral region	3.53	0.00	0.00	0.00
3	peripheral region	14.65	0.03	0.00	0.00
4	peripheral region	4.13	0.00	0.00	0.00
5	peripheral region	8.34	0.26	0.04	0.00
6	deep central region	11.04	0.23	0.01	0.00
7	deep central region	20.76	15.26	0.11	0.00
8	peripheral region	24.70	0.13	0.00	0.00
9	deep central region	28.65	99.95	39.61	0.63
Average			12.87 ± 33.04	4.42 ± 13.20	0.07 ± 0.21

**Table 5:** The TCP and NTCP for dose de-escalation in patient #7, and #9.

Patient	Probability	1800cGy/1F			1500cGy/1F		1400cGy/1F		1000cGy/1F	
		VMAT	IMPT	SPArc	VMAT	IMPT	VMAT	IMPT	VMAT	IMPT
#7	TCP(%)	97.45	96.00	97.29	93.96	90.72	91.77	87.50	69.95	59.30
	NTCP(%)	15.26	0.11	0.00	0.85	0.00	0.25	0.00	0.00	0.00
#9	TCP(%)	96.20	96.18	97.08	91.17	91.09	88.09	87.97	60.58	60.20
	NTCP(%)	99.95	39.61	0.63	80.33	2.82	54.42	0.81	0.60	0.00

Lastly, to test if the decision tree would help select the optimal plan from a clinician’s point of view, six physicians from different institutions worldwide voluntarily participated in this blind study to evaluate the dosimetric plan quality and preference. The result showed good agreement compared to the decision tree, along with some interesting findings. All the physicians selected the SPArc plan (#c) as the optimal solution for the single lesion brain SRS, even though the VMAT plan of patients #1 and #2 offered a slightly better CI and R50 than SPArc due to the target location and sizes (**Figure S2**). The physicians preferred a better mean dose sparing to the healthy brain tissue when the plans’ CI and R50 are comparable but not significantly inferior (**Figure S3**).

## Discussion

This study explored the dosimetric features of using the state-of-the-art proton beam therapy technique - IMPT and a new treatment modality - SPArc compared with the reference planning group, VMAT, in single lesion brain SRS. Since the clinical scenarios are very complicated because of the various size of targets and locations, additional time and resources are often needed to generate comparison plans for each patient to justify the benefit of using proton beam therapy. The brain SRS dosimetric model built in this study could assist the clinical decision among IMPT, VMAT, and SPArc techniques. It is also one major step forward since the publication of VMAT for brain SRS model reported by Atkins et al. in 2018 as proton beam therapy becomes more accessible (5). More interestingly, six out of six physicians from different institutions worldwide selected the SPArc plan over the VMAT plan in case #1, where small size targets (1.66 cc) were located in the deep central location, although the VMAT plan offered slightly better V_12Gy_ (3.55 cc) compared SPArc (4.06 cc). In this situation, when the dose conformity is comparable and not significantly different, the physicians considered the mean dose sparing for normal brain as a factor in the selection of the treatment modality, even though the relationship of cognitive function impairment with low radiation dose to the normal brain tissue is still under investigation (38). In the supplemental document, eleven additional patients with single brain metastasis were retrospectively included in the study. The target volumes, previous clinical prescribed doses (Gy), dosimetric metrics, and delivery time were summarized in **Tables S2**, **S3**, **S4**, and **Figures S4**, **S5**. The results also showed good agreement between the in silico brain SRS model and clinical cases.

Our study shows that the dosimetric metrics are critical when using the SPArc technique because a sharp dose fall-off and high dose sparing of the healthy brain tissue are clinically desired in brain SRS. In addition to the dosimetric metrics index comparison, the probability of brain RN was estimated based on the NTCP model. The radiation-induced brain necrosis was less than 1% in the majority of patients except patient #7 (15.26% with target volume 20.76 cc (D = 3.4cm)) and #9 (99.95% with target volume 28.65 cc (D =3.8 cm)) in VMAT planning group. The finding was consistent with the inverse relationship between the SRS dose and treatment volume and location with regard to the incidence of brain RN (35, 39, 40). It is worth noting that SPArc could reduce the risk of brain necrosis to less than 1% for all the patients with prescription dose 18 Gy in 1 fraction in these challenging situations, which indicated its potential clinical role in the management of a single brain lesion with large volume.

There are few reports on the brain SRS using the IMPT technique due to the large lateral penumbra. In 2014 and 2015, Hyer at al. and Wang et al. (41–43) discussed the limitation of using IMPT for peripheral single-target brain SRS compared to the VMAT. Thus, they introduced the aperture concept in PBS to sharpen the lateral penumbra. Our findings agreed with theirs, motivating us to investigate SPArc as the new treatment modality for this disease site. The results also indicated that a lower prescription is needed to mitigate such risk using IMPT or VMAT technique for single fractionated brain SRS (40). However, dose de-escalation will compromise the target local control, accessed based on the logistic tumor control probability (TCP) model and the Poission TCP model (36, 44, 45). The results suggest that TCP would have been significantly compromised in order to achieve a similar risk of brain RN as a SPArc plan (**Table 5**). More specifically, for patient #9, the single fraction dose in VMAT and IMPT had to be reduced to 10 Gy and 14 Gy, respectively, in order to achieve less than 1% of brain RN. This prescription dose level represents 60.58% and 87.97% TCP via VMAT and IMPT plan, respectively.

The use of range shifters (RS) in treating superficial targets sometimes complicates the clinical workflow e.g., clearance check with patient body and move in/out the range shifter. Additionally, it also introduces secondary proton scattering from the RS itself, which increases spot size when entering the patient’s body resulting in an inferior treatment plan quality due to the larger lateral penumbra. This study demonstrated that SPArc does not need to use RS even for the peripheral target while providing a superior dosimetric plan quality, simplifying the clinical workflow with a practically achievable treatment delivery time compared to the current standard-of-care IMPT.

The results from this study showed that the VMAT/VMAT(FFF) has, in general, the longer treatment delivery time compared to the SPArc treatment technique. Three-field IMPT shows its efficiency in the treatment delivery for single lesion brain SRS at the cost of treatment plan quality compared to SPArc. However, it is important to mention that the estimated treatment delivery time listed in **Table 2** only includes beam-on and gantry rotational times. The beam request time and treatment field loading time from Oncology Information System (OIS), e.g., ARIA or MOSIAQ to the proton delivery system, were not taken into account. In other words, additional time might be needed in the multi-field clinical IMPT workflow. This is one of the motivations why the proton treatment technique is moving towards the arc approach which not only has the potential to improve the plan quality but also simplify the clinical treatment workflow. Furthermore, it has been reported that the ELST could achieve 0.2 s in a cyclotron accelerator energy selection system (46). Such ELST technique and engineering advancements would result in a more efficient SPArc treatment in future clinical implementation.

This study explored the feasibility of utilizing SPArc for single brain metastases treatment. However, please note that such a novel technique is currently in the research and development stage, requiring the upgrades of the existing proton therapy systems, introducing new hardware and software, and incorporating the concept into the commercial treatment planning system. As a result, it may take years of technical and engineering development in order to implement it into routine clinical practice. Besides the SPArc technique, other existing technologies could be an option in brain radiosurgery, such as dynamic collimator system (41) and passive scattering with a unique patient immobilization system (47). Many of these technological limitations are advancing and the manufacturing time for patient devices (apertures and compensators) can be minimized to generate a plan efficiently. Boczkowskiet et al. determined the optimal plan parameters (define the aperture with a tight margin of 0.5mm and use of compensators to better shape distally) on a single metastatic lesion by comparing the proton SRS and photon VMAT SRS treatment plans (48, 49). Righetto et al. investigated the influence of spot spacing, apertures, and the margin from the CTV on the plan quality in treating neuromas and meningiomas (50). Recently, Atkins et al. reported the retrospective study of 370 patients treated using passive-scattering system. The local control rate and toxicity were found comparable to the conventional photon technique (5). It would be interesting to compare these two merging techniques, SPArc and aperture-based IMPT, to further explore the dosimetric plan quality, especially in the target conformity, low dose sparing, and treatment delivery efficiency. With all of the ongoing developments, it is optimistic that proton beam therapy will become a growing treatment option for brain SRS.

Several points require further discussion. First, we acknowledge that the SPArc for brain tumors is not performed in routine clinical practice; however, our study was designed as a proof-of-principle to determine clinical scenarios in which protons may have the chance to offer superior brain sparing compared to the conventional VMAT. To achieve this, we attempted to equilibrate as many treatment planning variables as possible between the three modalities. Second, the model did not include the brainstem in the consideration, so it may not be suitable for the clinical situation where the target is inside or abutting the brainstem.

In summary, this study validated the brain SRS dosimetric model using nine clinical cases and a blind survey. For the cancer centers equipped with both photon and proton treatment facilities, this model-based decision tree provides a practical tool as *a priori* knowledge in selecting IMPT, VMAT, or SPArc without generating a comparable plan, which has the potential to reduce the planning workload and improve clinical workflow efficiency.

## Conclusions

At the current stage of technological development, VMAT holds the dosimetric advantage in the single brain lesion SRS over IMPT. With the new technology, SPArc showed its potential clinical advantage to offer lower dose to the brain tissue over VMAT with an equivalent or higher CI in the peripheral brain lesion and deep centrally located lesion larger than 9 cc. The brain SRS dosimetric model developed in this study could be used as a future reference tool to assist the clinical decision in selecting the optimal treatment modality for the patient.

## Data Availability Statement

The raw data supporting the conclusions of this article will be made available by the authors, without undue reservation.

## Ethics Statement

The study has been approved by Beaumont Health Internal Review Board #2017-455. Written informed consent for participation was not required for this study in accordance with the national legislation and the institutional requirements. Written informed consent was not obtained from the individual(s) for the publication of any potentially identifiable images or data included in this article.

## Author Contributions

SC and GL contributed to the acquisition, analysis, and interpretation of data and drafted and designed the paper. LZ, WZ, and DY provided technical support. WZ, DY, PChc, XPL, KY, RD, CS, IG and PChi contributed to revising the paper and providing clinical inputs. XQL provided physics and clinical support. XD contributed to the design of the study, revised the draft, and led the research direction. All authors contributed to the article and approved the submitted version.

## Funding

The study is supported by Ion Beam Application S.A. (IBA, Belgium) and Beaumont health seed grant award. The funders were not involved in the study design, collection, analysis, interpretation of data, the writing of this article, or the decision to submit it for publication.

## Conflict of Interest

This research was supported by Beaumont Research Seed Grant Award and Ion Beam Application. XD received honorarium from IBA Speaker Bureau outside the work presented in this study.

The remaining authors declare that the research was conducted in the absence of any commercial or financial relationships that could be construed as a potential conflict of interest.

## Publisher’s Note

All claims expressed in this article are solely those of the authors and do not necessarily represent those of their affiliated organizations, or those of the publisher, the editors and the reviewers. Any product that may be evaluated in this article, or claim that may be made by its manufacturer, is not guaranteed or endorsed by the publisher.
