# Supplementary material for: Redefine the Role of Spot-Scanning Proton Beam Therapy for the Single Brain Metastasis Stereotactic Radiosurgery
Source: Front Oncol. 2022 May 19;12:804036. doi: 10.3389/fonc.2022.804036 (PMC9160604; doi:10.3389/fonc.2022.804036)
Supplement: Supplementary file 1 [file DataSheet_1.docx]

**Supplemental document**

**Supplementary Table 1.**

The beam angle selection of different modalities.

| Modality | Beam angle | | | | |
| --- | --- | --- | --- | --- | --- |
| VMAT | 4 arcs | 181° to 179° (couch=0° ) | 179° to 181° (couch=0° ) | 181° to 0° (couch=45° ) | 0° to 179° (couch=315° ) |
| SPArc | 3 arcs | 181° to 179° (couch=0° ) | 181° to 0° (couch=45° ) | 0° to 179° (couch=315° ) |  |
| IMPT | 3 beams | Beam angle (couch=0°) | Beam angle (couch=0°) | Beam angle 90° (couch=270°) |  |


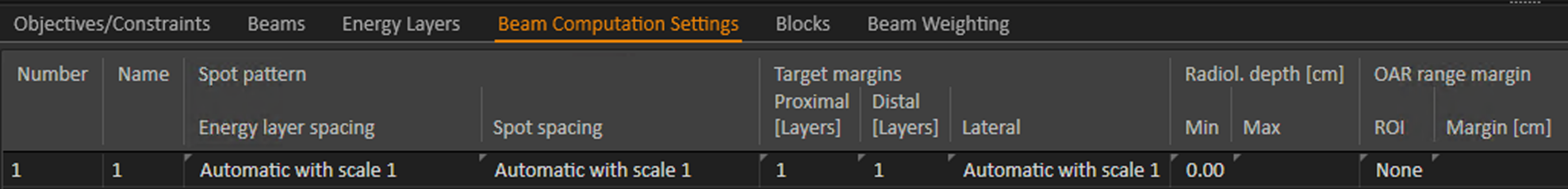


**Supplementary Figure 1.** RayStation’s default parameter such as the automatic energy layer spacing and spot spacing for optimization.


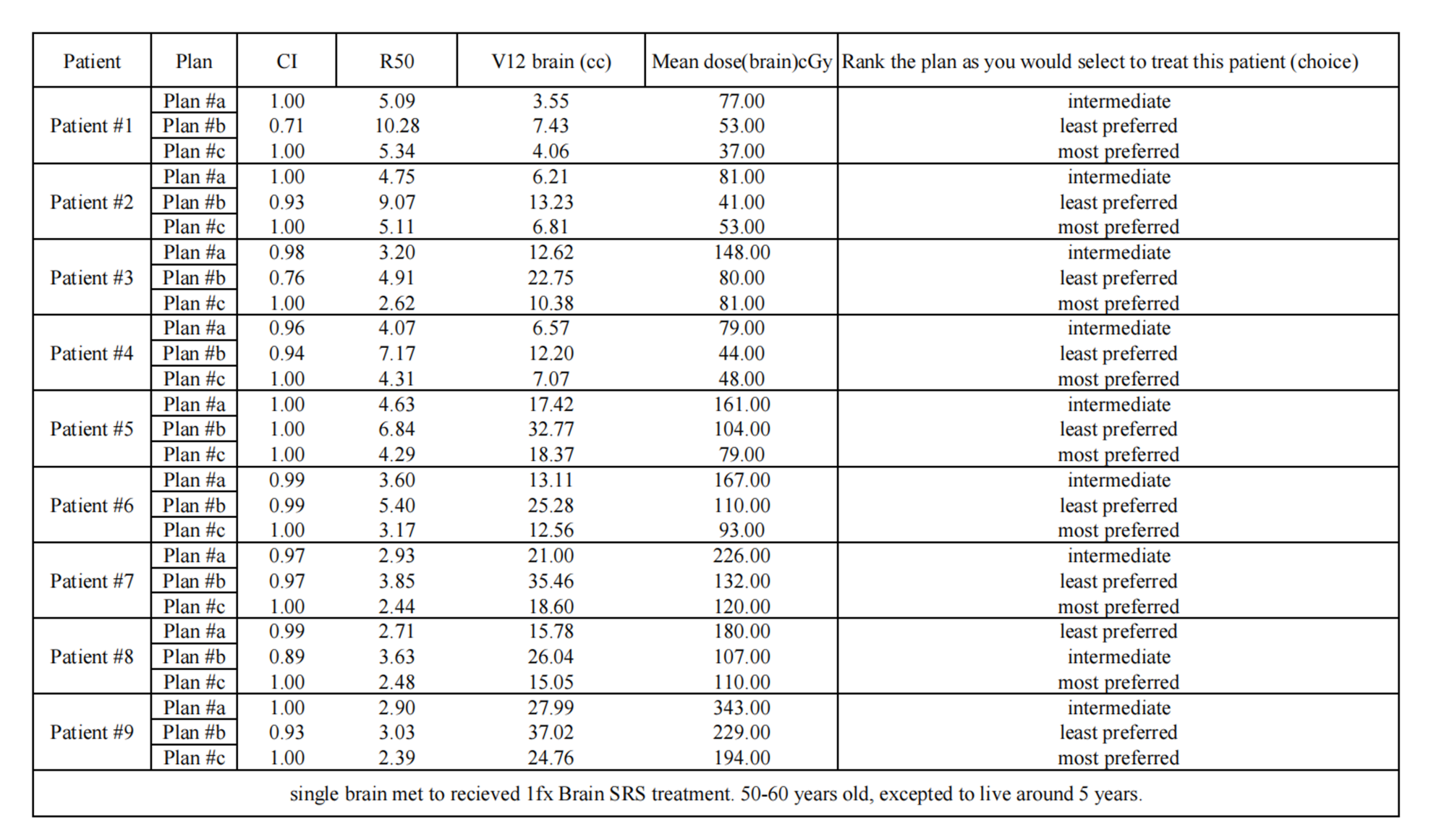


**Supplementary Figure 2.** An example of the physician’s survey. The most preferred choice, the intermediate choice, the least preferred choice for the three planning groups.

**
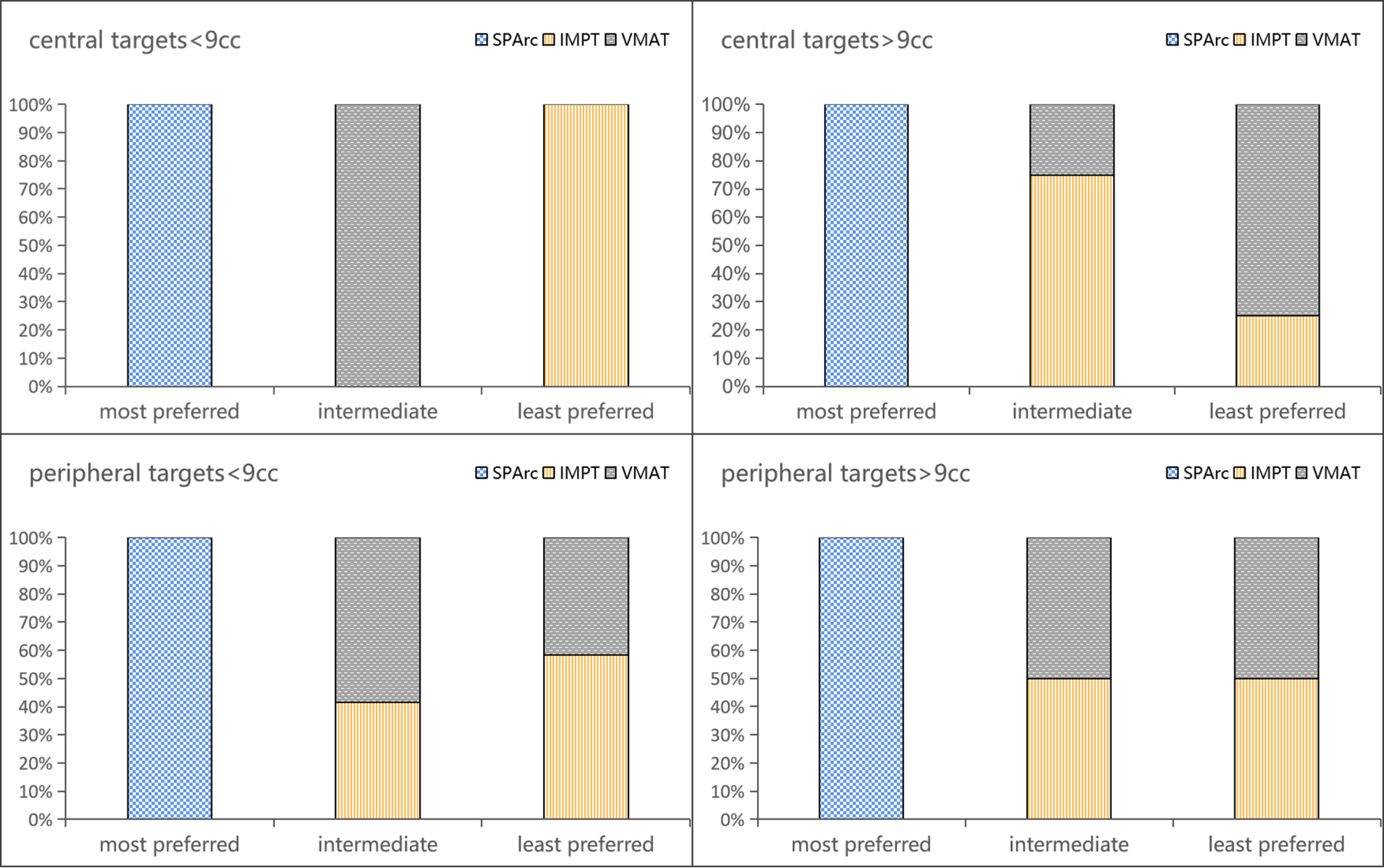
Supplementary Figure 3.** The stacked percentage columns of physician’s blinded survey, where each column totals 100%. The most preferred choice, the intermediate choice, the least preferred choice for the three planning groups: SPArc, VMAT and IMPT at deep central and peripheral region of different target sizes.

To validate the *in silico* brain SRS model, more eleven patients with single brain metastasis were retrospectively included in the study. The target volumes and previous clinical prescribed doses (Gy) and fraction are detailed in Table s2. These eleven cases were separated into two groups based on the location: five deep central and six peripheral located targets. The GTVs volume ranges from 3.14 cc to 18.11 cc. The target CI, R50, mean dose of the brain, and V_12Gy_ of the cases were analyzed and compared to the brain SRS dosimetric model (Fig. s4, 5 and Table s4). The results also showed a good agreement between the *in silico* model and the clinical cases. Both SPArc and IMPT planning groups reduced the mean brain dose delivered to all patients compared to the VMAT planning group. In terms of the delivery time (including irradiation time, gantry rotation, and couch rotation), VMAT and VMAT (FFF) plans took much longer to deliver than any proton-based radiosurgery plan (Table s3).

**Supplementary Table 2.**

Target size and previous clinical prescription for the twenty patients included in this SRS study comparison.

| Patient | Tumor location | Tumor volume (cc) | Previous Clinical prescription dose and fraction |
| --- | --- | --- | --- |
| #1 | deep central region | 1.66 | 21Gy/1f |
| #2 | peripheral region | 3.53 | 21Gy/1f |
| #3 | peripheral region | 14.65 | 15Gy/1f |
| #4 | peripheral region | 4.13 | 18Gy/1f |
| #5 | peripheral region | 8.34 | 18Gy/1f |
| #6 | deep central region | 11.04 | 15Gy/1f |
| #7 | deep central region | 20.76 | 24Gy/3f |
| #8 | peripheral region | 24.70 | 15Gy/1f |
| #9 | deep central region | 28.65 | 21Gy/3f |
| #10 | deep central region | 3.14 | 21Gy/1f |
| #11 | deep central region | 5.89 | 21Gy/1f |
| #12 | deep central region | 7.25 | 18Gy/1f |
| #13 | deep central region | 9.83 | 15Gy/1f |
| #14 | deep central region | 14.83 | 15Gy/1f |
| #15 | peripheral region | 1.13 | 24Gy/1f |
| #16 | peripheral region | 5.87 | 18Gy/1f |
| #17 | peripheral region | 7.08 | 18Gy/1f |
| #18 | peripheral region | 12.80 | 15Gy/1f |
| #19 | peripheral region | 15.57 | 15Gy/1f |
| #20 | peripheral region | 18.11 | 15Gy/1f |

**Supplementary Table 3.**

Comparison of total delivery time per patient’s SRS plan.

| Delivery time (Patient#) | Tumor volume (cc) | VMAT  (600 MU/min, s) | VMAT[1-5]  (FFF, 1400 MU/min, s) | IMPT (s) | SPArc (s) |
| --- | --- | --- | --- | --- | --- |
| 1 | 1.66 | 1635 | 808 | 85 | 207 |
| 2 | 3.53 | 1340 | 851 | 128 | 240 |
| 3 | 14.65 | 1516 | 972 | 206 | 288 |
| 4 | 4.13 | 1369 | 807 | 141 | 217 |
| 5 | 8.34 | 1946 | 1012 | 193 | 251 |
| 6 | 11.04 | 1809 | 1034 | 172 | 238 |
| 7 | 20.76 | 1631 | 1034 | 214 | 259 |
| 8 | 24.70 | 1605 | 1061 | 275 | 313 |
| 9 | 28.65 | 1322 | 889 | 244 | 328 |
| 10 | 3.14 | 1473 | 628 | 84 | 202 |
| 11 | 5.89 | 2131 | 885 | 101 | 245 |
| 12 | 7.25 | 2330 | 963 | 115 | 241 |
| 13 | 9.83 | 2172 | 914 | 136 | 301 |
| 14 | 14.83 | 1831 | 774 | 139 | 305 |
| 15 | 1.13 | 1324 | 570 | 108 | 166 |
| 16 | 5.87 | 1577 | 671 | 155 | 241 |
| 17 | 7.08 | 1726 | 729 | 126 | 262 |
| 18 | 12.80 | 1958 | 820 | 187 | 346 |
| 19 | 15.57 | 1787 | 769 | 165 | 334 |
| 20 | 18.11 | 2335 | 985 | 200 | 372 |
| Average | | 1740.85±322.95 | 858.80±141.58 | 158.70±52.35 | 267.80±53.75 |
| P | | - | <0.01* | <0.01* | <0.01* |

*P＜0.05 while comparing the VMAT plan with other two plans.

**Supplementary Table 4.**

Dosimetric metrics among VMAT, IMPT and SPArc for the twenty patients.

| patient | Tumor location | Tumor volume  (cc) | CI | | | R50 | | | | | V_12Gy_(brain) (cc) | | | | | Mean dose(brain) (cGy) | | | | |
| --- | --- | --- | --- | --- | --- | --- | --- | --- | --- | --- | --- | --- | --- | --- | --- | --- | --- | --- | --- | --- |
|  |  |  | VMAT | IMPT | SPArc | VMAT | Relative to SPArc | IMPT | Relative to SPArc | SPArc | VMAT | Relative to SPArc | IMPT | Relative to SPArc | SPArc | VMAT | Relative to SPArc | IMPT | Relative to SPArc | SPArc |
| 1 | deep central | 1.66 | 1.00 | 0.99 | 1.00 | 5.09 | 8.97 | 9.15 | 16.12 | 5.34 | 3.55 | 7.10 | 7.43 | 14.87 | 4.06 | 77.00 | 122.97 | 50.00 | 79.85 | 37.00 |
| 2 | peripheral region | 3.53 | 1.00 | 0.93 | 1.00 | 4.75 | 5.21 | 9.07 | 9.94 | 5.11 | 6.21 | 8.63 | 13.23 | 18.38 | 6.81 | 81.00 | 87.62 | 41.00 | 44.35 | 53.00 |
| 3 | peripheral region | 14.65 | 0.98 | 0.93 | 1.00 | 3.20 | 3.81 | 4.05 | 4.82 | 2.62 | 12.62 | 22.71 | 16.61 | 29.89 | 10.38 | 148.00 | 199.87 | 68.00 | 91.83 | 81.00 |
| 4 | peripheral region | 4.13 | 0.96 | 0.94 | 1.00 | 4.07 | 4.77 | 7.17 | 8.40 | 4.31 | 6.57 | 9.46 | 12.20 | 17.57 | 7.07 | 79.00 | 100.58 | 44.00 | 56.02 | 48.00 |
| 5 | peripheral region | 8.34 | 1.00 | 1.00 | 1.00 | 4.63 | 4.12 | 6.84 | 6.09 | 4.29 | 17.42 | 13.56 | 32.77 | 25.51 | 18.37 | 161.00 | 172.33 | 104.00 | 111.32 | 79.00 |
| 6 | deep central | 11.04 | 0.99 | 0.98 | 1.00 | 3.60 | 4.00 | 4.84 | 5.37 | 3.17 | 13.11 | 14.46 | 21.17 | 23.34 | 12.56 | 167.00 | 208.53 | 102.00 | 127.37 | 93.00 |
| 7 | deep central | 20.76 | 0.97 | 0.94 | 1.00 | 2.93 | 3.29 | 3.53 | 3.96 | 2.44 | 21.00 | 19.75 | 31.04 | 29.19 | 18.60 | 226.00 | 289.62 | 127.00 | 162.75 | 120.00 |
| 8 | peripheral region | 24.7 | 0.99 | 0.99 | 1.00 | 2.71 | 2.85 | 3.48 | 3.66 | 2.48 | 15.78 | 23.96 | 23.28 | 35.35 | 15.05 | 180.00 | 227.05 | 101.00 | 127.40 | 110.00 |
| 9 | deep central | 28.65 | 1.00 | 0.93 | 1.00 | 2.90 | 3.08 | 3.03 | 3.22 | 2.39 | 27.99 | 25.25 | 37.02 | 33.40 | 24.76 | 343.00 | 339.78 | 229.00 | 226.85 | 194.00 |
| 10 | deep central | 3.14 | 1.02 | 0.94 | 1.00 | 5.48 | 5.29 | 8.50 | 8.20 | 6.50 | 7.40 | 7.05 | 14.20 | 13.52 | 10.40 | 92.00 | 110.34 | 68.00 | 81.56 | 61.00 |
| 11 | deep central | 5.89 | 1.04 | 0.90 | 1.00 | 5.25 | 5.65 | 5.76 | 6.20 | 4.23 | 12.70 | 12.39 | 16.80 | 16.38 | 11.40 | 115.00 | 147.29 | 83.00 | 106.30 | 68.00 |
| 12 | deep central | 7.25 | 1.01 | 0.94 | 1.00 | 4.40 | 5.28 | 5.05 | 6.06 | 3.41 | 12.80 | 13.85 | 18.10 | 19.59 | 11.10 | 138.00 | 183.16 | 88.00 | 116.80 | 72.00 |
| 13 | deep central | 9.83 | 1.01 | 0.96 | 1.00 | 3.48 | 4.13 | 4.13 | 4.90 | 3.12 | 13.50 | 14.00 | 18.90 | 19.60 | 12.80 | 147.00 | 214.78 | 90.00 | 131.50 | 75.00 |
| 14 | deep central | 14.83 | 1.04 | 0.96 | 1.00 | 3.29 | 3.91 | 3.67 | 4.36 | 2.60 | 18.10 | 18.08 | 23.30 | 23.28 | 15.00 | 176.00 | 246.84 | 101.00 | 166.20 | 79.00 |
| 15 | peripheral region | 1.13 | 0.95 | 0.75 | 1.00 | 10.44 | 8.84 | 23.81 | 20.16 | 11.06 | 5.40 | 4.49 | 14.20 | 11.80 | 6.40 | 68.00 | 78.63 | 38.00 | 43.94 | 32.00 |
| 16 | peripheral region | 5.87 | 1.00 | 0.94 | 1.00 | 6.01 | 5.03 | 8.62 | 7.21 | 5.28 | 12.90 | 11.59 | 21.70 | 19.49 | 13.30 | 114.00 | 142.89 | 53.00 | 66.43 | 57.00 |
| 17 | peripheral region | 7.08 | 1.01 | 0.96 | 1.00 | 4.68 | 4.93 | 5.73 | 6.03 | 3.83 | 14.70 | 15.22 | 20.00 | 20.71 | 12.70 | 109.00 | 158.62 | 64.00 | 93.14 | 54.00 |
| 18 | peripheral region | 12.80 | 0.94 | 0.89 | 1.00 | 4.56 | 4.29 | 5.02 | 4.72 | 3.49 | 17.70 | 21.86 | 22.10 | 27.30 | 14.40 | 104.00 | 210.35 | 48.00 | 97.08 | 51.00 |
| 19 | peripheral region | 15.57 | 1.03 | 0.99 | 1.00 | 3.30 | 3.52 | 3.72 | 3.97 | 2.79 | 19.10 | 21.24 | 23.90 | 26.58 | 17.20 | 112.00 | 247.06 | 63.00 | 138.97 | 51.00 |
| 20 | peripheral region | 18.11 | 1.04 | 0.94 | 1.00 | 3.32 | 3.16 | 4.15 | 3.94 | 3.01 | 21.40 | 21.08 | 29.80 | 29.35 | 20.60 | 143.00 | 257.23 | 72.00 | 129.51 | 67.00 |

CI: Conformity Index, (Ratio of the target volume to 100% Prescription isodose volume);

R50: (Ratio of 50% Prescription isodose volume to the target volume);

V_12_Gy: (Volume of brain tissue minus GTV receiving12 Gy).

Relative to SPArc (model): (Ratio of SPArc plan between the model and case) * (absolute dosimetric metrics).


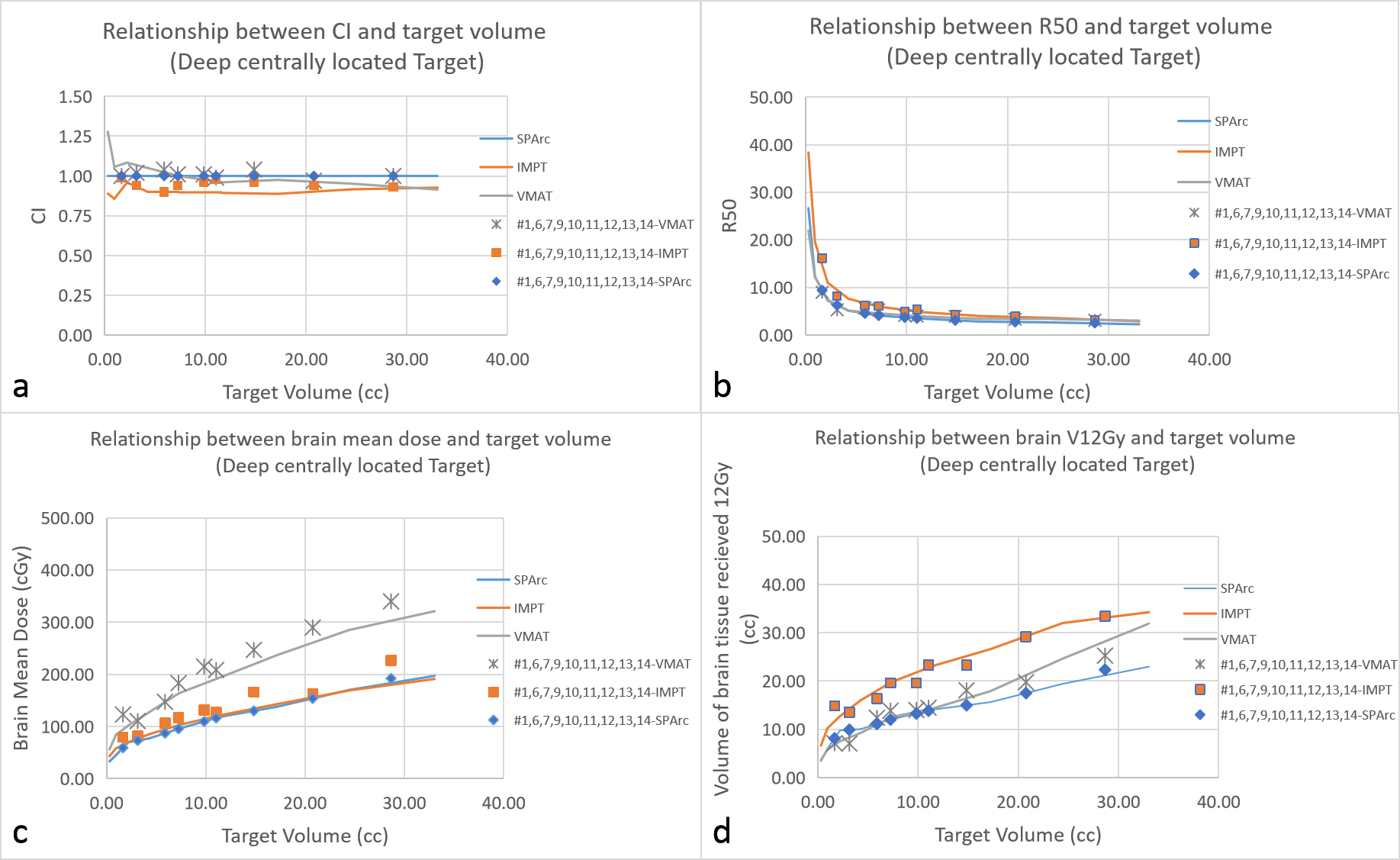


**Supplementary Figure 4.** Dosimetric metrics among three planning groups: SPArc, VMAT, and IMPT at deep central (a, b, c, d) of different target sizes. Dots, squares, and stars are the dosimetric metrics extracted from twenty clinical validation cases normalized to SPArc plan.


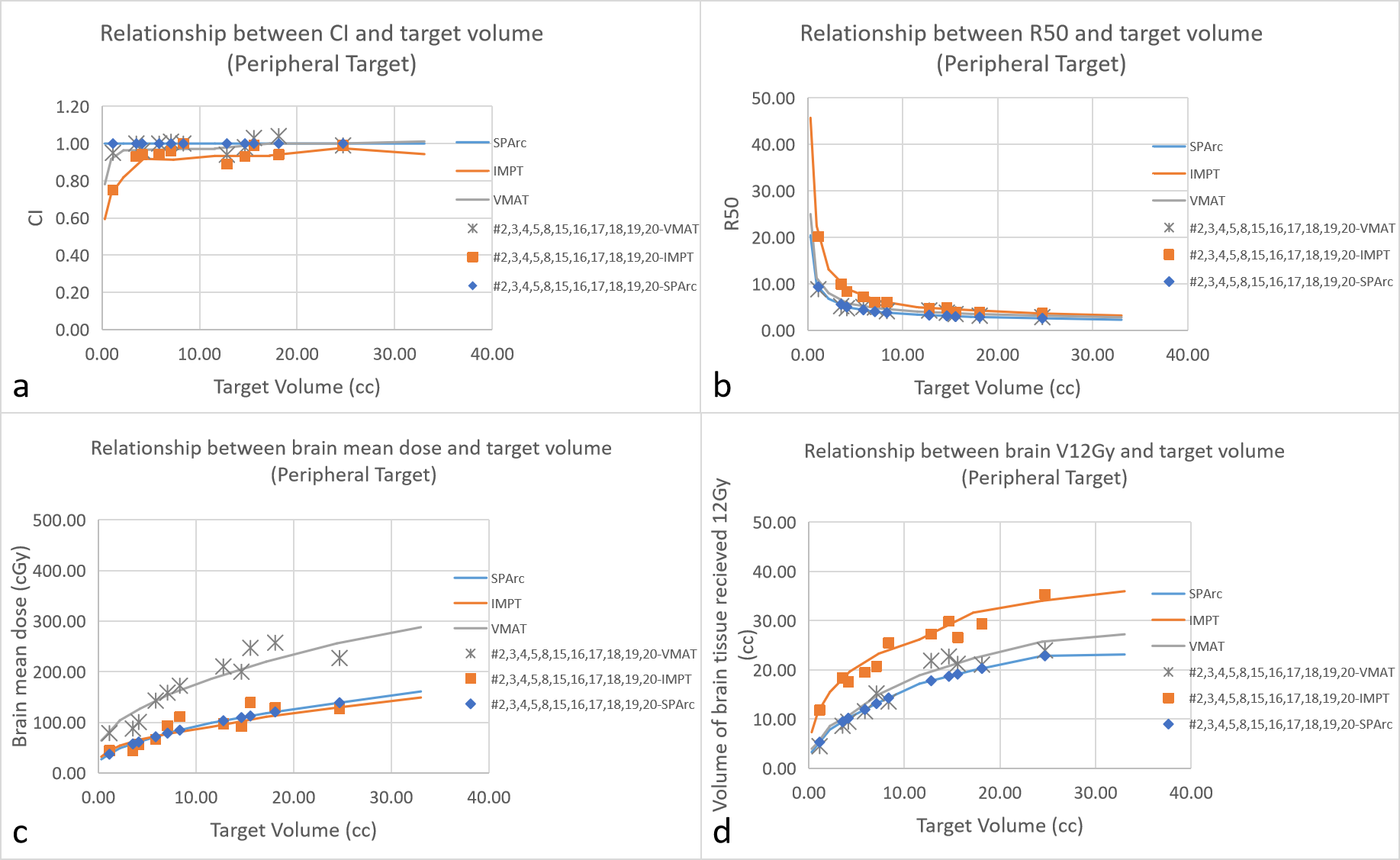


**Supplementary Figure 5.** Dosimetric metrics among three planning groups: SPArc, VMAT, and IMPT at peripheral region (a, b, c, d) of different target sizes. Dots, squares, and stars are the dosimetric metrics extracted from twenty clinical validation cases normalized to SPArc plan.
